# Producing recombinant proteins in *Vibrio natriegens*

**DOI:** 10.1186/s12934-024-02455-5

**Published:** 2024-07-24

**Authors:** Matthew Smith, José Sánchez Hernández, Simon Messing, Nitya Ramakrishnan, Brianna Higgins, Jennifer Mehalko, Shelley Perkins, Vanessa E. Wall, Carissa Grose, Peter H. Frank, Julia Cregger, Phuong Vi Le, Adam Johnson, Mukul Sherekar, Morgan Pagonis, Matt Drew, Min Hong, Stephanie R. T. Widmeyer, John-Paul Denson, Kelly Snead, Ivy Poon, Timothy Waybright, Allison Champagne, Dominic Esposito, Jane Jones, Troy Taylor, William Gillette

**Affiliations:** 1 Protein Expression Laboratory, Frederick National Laboratory for Cancer Research, Frederick, MD 21702 USA; 2 NCI RAS Initiative, Frederick National Laboratory for Cancer Research, Frederick, MD 21702 USA

**Keywords:** *Vibrio natriegens*, recombinant protein expression, *Escherichia coli*, auto-induction, protein aggregation, protein folding, TEV protease, isotopic labeling, small GTPase, KRAS4b

## Abstract

The diversity of chemical and structural attributes of proteins makes it inherently difficult to produce a wide range of proteins in a single recombinant protein production system. The nature of the target proteins themselves, along with cost, ease of use, and speed, are typically cited as major factors to consider in production. Despite a wide variety of alternative expression systems, most recombinant proteins for research and therapeutics are produced in a limited number of systems: *Escherichia coli*, yeast, insect cells, and the mammalian cell lines HEK293 and CHO. Recent interest in *Vibrio natriegens* as a new bacterial recombinant protein expression host is due in part to its short doubling time of ≤ 10 min but also stems from the promise of compatibility with techniques and genetic systems developed for *E. coli*. We successfully incorporated *V. natriegens* as an additional bacterial expression system for recombinant protein production and report improvements to published protocols as well as new protocols that expand the versatility of the system. While not all proteins benefit from production in *V. natriegens*, we successfully produced several proteins that were difficult or impossible to produce in *E. coli*. We also show that in some cases, the increased yield is due to higher levels of properly folded protein. Additionally, we were able to adapt our enhanced isotope incorporation methods for use with *V. natriegens*. Taken together, these observations and improvements allowed production of proteins for structural biology, biochemistry, assay development, and structure-based drug design in *V. natriegens* that were impossible and/or unaffordable to produce in *E. coli*.

## Highlights

Production of proteins with reduced aggregation compared to *E. coli*.

Optimized protocols for efficiency and protein expression.

Improved yield for specific proteins compared to *E. coli*.

## Background

It has long been established that recombinant protein expression platforms have advantages and deficiencies specific to the system. Accordingly, multiple systems have been developed with advantages and disadvantages and scientists use the system(s) that best meets their needs. For example, the often-cited advantages of *Escherichia coli* are low cost, speed, and the ease of genetic manipulation [1, 2]. Yet, protein production is often limited in *E. coli* due to issues in the areas of protein expression, solubility, protein folding, and activity, particularly when expressing large and/or eukaryotic proteins [3]. Nevertheless, *E. coli* is typically one of the first choices in a recombinant protein production effort for many research, biotechnology, and pharmaceutical laboratories, due to its many advantages. It is often the only bacterial option that laboratories consider, whereas there are multiple eukaryotic expression systems to choose from (e.g. yeast, insect/baculovirus, HEK293, and CHO) [3].

Thus, the development of *V. natriegens* as a bacterial expression system by Weinstock et al. [4], was an inflection point in recombinant protein expression research and many reports have followed, exploring the system for a variety of applications [5–9]. Until this recent increase in interest, the fast growth rate of *V. natriegens* [10] was one of the more notable attributes of this Gram-negative bacterium that was isolated from a salt marsh [11]. Reports have attributed this fast-growing phenotype to the large number of rRNA operons, and thus ribosomes [4, 12, 13], or a higher level of substrate uptake [14]. Regardless, *V. natriegens* might have some advantages over *E. coli* in recombinant protein expression.

The recent work cites fast growth rate, metabolic diversity, and the emerging development of tools for genetic manipulation as positive factors for the use of *V. natriegens* [15, 16]. Fast growth rate was also what induced our lab to investigate the use of this system for recombinant protein production. Yet, while we have found the fast growth rate to be useful, perhaps more impactful to our work has been our discovery that *V. natriegens* serves as a complementary expression system, allowing production of a subset of proteins that we were unable to easily produce in *E. coli* [17–19]. The roadblocks to production in *E. coli* that were overcome by switching to *V. natriegens*, suggest some fundamental difference(s) in the process between the two systems, e.g. protein folding via chaperones. While adopting *V. natriegens* as a parallel bacterial expression platform to our customized *E. coli* system [20], we encountered and overcame several obstacles that we report here. The protocols we developed are easy to implement, do not involve costly reagents or equipment, and expand the capabilities of *V. natriegens* as a practical addition to the field of bacterial recombinant protein expression.

## Methods

### Cloning, bacterial strains, and genetic resources

DNA constructs for the expression of Hs.KRAS4b(1-169), Hs.KRAS4b(2-169), Hs.NRAS(1-169), and Hs.RAF1(52–192) in the format of His6-MBP-tev-POI (MBP, maltose-binding protein; tev, tobacco etch virus protease recognition sequence; POI, protein of interest) were created by subcloning Entry Clones into pDest-566 (Addgene #11,517) using Gateway LR clonase per the manufacturer’s instructions (Thermo Fisher Scientific). Plasmid R714-X01 (renamed from NCBI Reference Sequence: NM_144547.2; Uniprot Q8K592; ref. 12), encoding FLAG-Mm.AMHR2(18–142)-His6, was received from the legacy Tuohy laboratory at the Cleveland Clinic. Nanobody expression plasmids were provided by Matt Hall (National Center for Advancing Translational Research (NCATS), Rockville, MD). See Table 1 for details of the proteins encoded by the plasmids used in this work.

*V. natriegens* was obtained from Synthetic Genomics, Inc. (now obtainable from Telesis Bio, San Diego). *E. coli* expression work was performed with a variant of strain BL21 DE3 Star™ (Thermo Fisher Scientific (Waltham, MA)) harboring pRare plasmid (Cm^R^) expressing tRNAs (argU, argW, ileX, glyT, leuW, proL, metT, thrT, tyrU and thrU). Our variant *E. coli* strain (BL21 DE3 TT1) is derived from BL21 DE3 Star™ as an isolate that is resistant to a bacteriophage discovered in our lab. It was isolated from bacterial colonies that grew on an agar plate spread with the bacteriophage.

The *V. natriegens* genomic sequence used for homology searches was NCBI RefSeq Assembly GCF_001456255.1. This sequence was determined from a strain of *V. natriegens* referred to as ATCC 14,048, DSM 759, or *Vibrio natriegens* NBRC 15,636.

### Chemicals and media

Unless noted otherwise, all chemicals were obtained from MilliporeSigma (Burlington, MA). Instant Ocean™ was from Spectrum Brands (Blacksburg, VA). DMSO was from NEB (Ipswich, MA). Brain Heart Infusion (BHI) Dry media was from Thermo Fisher Scientific. BHI broth medium was prepared as per [4] (BHI + v2 salts) but without the addition of MgCl_2_ (referred to here as BHIv2-Mg). LB-15 agar petri plates were Lysogeny Broth (LB-Miller modified to 15 g/L NaCl) with 2.0% (w/v) agar amended as needed with either 5 µg/mL ampicillin (for initial transformation plates) or 50 µg/mL ampicillin (for colony isolation) for plasmid maintenance. All liquid cultures were amended with 50 µg/mL ampicillin. ZYM-20052 medium [20] was modified to 1.5% (w/v) Instant Ocean™ with no lactose added (referred to here as ZYM-20050-IO) and was used for overnight seed growths. TBV2 medium is (per liter) 12 g tryptone, 24 g yeast extract, 15 g NaCl, 0.5% (v/v) glycerol, 2.31 g KH_2_PO_4_, and 16.43 g K_2_HPO_4_⋅3H_2_O. The potassium salts are prepared separately in ~ 100 mL dH_2_O (per liter of final volume), filter sterilized, and added to the other components which had been autoclaved separately.

### Preparation of chemically competent cells

Chemically competent *V. natriegens* cells were prepared as described [4] by Weinstock et al., with modifications. The published *V. natriegens* protocol is a variation on the standard CCMB80 method originally developed by Hanahan for preparing *E. coli* competent cells [21], that incorporates media specific for *V. natriegens* and incorporates PIPES and DMSO into the transformation storage buffer. Modifications from the Weinstock protocol that we introduced were: (1) a glycerol stock was used to obtain an isolated colony by a T-streak on an LB-15 agar plate (no antibiotics) and incubated overnight at 30 °C, (2) MgCl_2_ was omitted from the BHI broth, and (3) the final pool of competent cells in transformation storage buffer (prepared as described [4]) was amended with filter sterilized glycerol to 10% (v/v).

### Transformation of chemically competent cells

A vial of *V. natriegens* competent cells was retrieved from storage at − 80 °C and allowed to thaw on ice. One hundred ng of plasmid DNA (isolated from *E. coli* DH10B T1 Phage-Resistant cells, Thermo Fisher Scientific) was added to the tube of competent cells, mixed by flicking the tube gently (~ 10 times), and incubated on ice for 30 min. During incubation, 1 mL BHIv2-Mg was added to a culture tube (14 mL round bottom Falcon™ tube, cat# 352059) and warmed to 30 °C. The cells/DNA mixture was heat shocked in a 42 °C water bath (without shaking) for 30 s and returned to ice for 1.5 min. The 1 mL of warmed BHIv2-Mg medium was added to the cell/DNA mixture, and the transformation reaction was transferred from the microcentrifuge tube back to the culture tube and incubated at 30 °C for 1 h with agitation at 250 rpm (1” throw). Three hundred microliters of the transformation were plated on antibiotic selection plates (LB-15 agar plate with 5 µg/mL ampicillin, pre-warmed to 30 °C) and incubated at 30 °C overnight. The next morning a colony was picked from the 5 µg/mL ampicillin plate and struck out on an LB-15 agar, 50 µg/mL ampicillin plate to obtain isolated colonies by T-streak and incubated at 30 °C. After ~ 6–8 h, colonies were visible.

### Small-scale growth

Fifty milliliters of ZYM-20050-IO with 50 µg/mL ampicillin in a baffled, 250 mL flask was inoculated with an ice chip from a *V. natriegens* glycerol stock. This seed culture was incubated at 30 °C for 14–16 h with shaking at 250 rpm (1” throw). The following morning, the OD_600_ of the overnight culture was measured and used to inoculate 30 mL of TBV2 medium + 50 µg/mL ampicillin in a 250 mL baffled flask (Optimum Growth™, Thomson, Oceanside, CA) to a calculated OD_600_ of 0.1. The flask was incubated at 30 °C, shaking at 250 rpm (1” throw). The culture was grown to an OD_600_ of 1.5-2.0 (~ 2 h) and protein expression induced with 1 mM of IPTG. The culture was grown for another 6–7 h and cells were harvested using a 50 mL conical tube at 3900 x *g* for 25 min at 4 °C. Fifty milliliter *E. coli* cultures were grown using the Dynamite medium protocol as described previously [20].

### Large-scale *V. natriegens* growth for protein production

To produce two liters of expression culture, a BioFlo 110 (3-liter vessel, 2-liter working volume, New Brunswick) was prepared the day before the planned growth with a dissolved oxygen (DO) probe (with fresh electrolyte) in two liters of TBV2 medium and autoclaved using a liquid 30 min cycle. The DO probe was attached to the head unit and allowed to polarize overnight (per manufacturer’s instructions). Fifty milliliters of ZYM-20050-IO in a 250 mL baffled flask was inoculated with an ice chip from a *V. natriegens* glycerol stock. This seed culture was incubated overnight at 30 °C and shaken at 250 rpm (1” throw). The following morning the TBV2 medium in the 3-liter production vessel was completed by adding 200 mL of filter sterilized potassium salts, antifoam (Antifoam 204, added at 0.5 mL/L from a 50% (v/v with water) stock), and 50 µg/mL ampicillin. The air flow was set to 3 L/min, the temperature set to 30 °C, and the agitation was set to 481 rpm. The minimum target DO was set to 20%, controlled by agitation primarily (agitation range was set to 481–600 rpm) and then by controlling supplied O_2_ (range set to 0-100%). The overnight culture density was measured by OD_600_ and used to inoculate the TBV2 medium in the BioFlo 110 to a calculated OD_600_ of 0.1. The culture was grown to an OD_600_ of 1.5-2 (~ 2 h), protein expression induced with 1 mM of IPTG, the culture grown for an additional ~ 4 h, final OD_600_ measured, and the culture harvested at 9000 x *g* for 30 min at 4 °C. When expression constructs included the CRD domain of RAF1, ZnCl_2_ was added at 300 µM approximately 1 h before induction of protein expression. Cell pellets were either lysed immediately or stored at -80 °C. Two-liter *E. coli* cultures were grown using the Dynamite medium protocol as described previously [20].

### ^15^N isotopic labeling of Hs.RAF1 (52–192)

Fifty milliliters of ZYM-20050-IO in a 250 mL baffled flask was inoculated with an ice chip from a *V. natriegens* glycerol stock. This seed culture was incubated overnight at 30 °C and shaken at 250 rpm (1” throw). The following morning, one liter of ModM9 [22] medium (amended to 4 g/L glucose, 15 g/L NaCl, and 50 mM ^15^NH_4_Cl, cat # NLM-467, Cambridge Isotope Laboratories, Inc., Tewksbury, MA) was prepared in a four-liter baffled shake flask. The OD_600_ of the overnight culture was measured and the amount of overnight culture required to achieve a starting OD_600_ of 0.1 was aseptically withdrawn, centrifuged (3900 x *g* for 15 min at room temperature), resuspended with a small amount of the prepared ModM9, added to the remaining ModM9 culture in the four-liter flask, and incubated at 30 °C, 250 rpm (1” throw), for a target OD_600_ of 0.5. At ~ 1 h prior to reaching that density, the culture was amended to 300 µM ZnCl_2_ (to assist with the folding of the cysteine rich domain). Upon reaching OD_600_ = 0.5, the protein expression was induced by the addition of 1 mM IPTG and shifted to 25 °C for overnight incubation. Cells were harvested by centrifugation (9000 x *g*, 30 min at 4 °C). The protocols for isotopic labeling in *E. coli* were described previously [23]. Briefly, an overnight culture was grown in MDAG medium [20], washed cells from this overnight were used to inoculate minimal Mod M9 media [22] containing ^15^N NH_4_Cl. At an OD_600_ of ~ 0.5, ZnCl_2_ was added to 300 µM (to assist with the proper folding of the cysteine rich domain), IPTG added at 0.5 mM, and the culture incubated for 3 h. All cell culture steps were performed at 37 °C. Cells were harvested by centrifugation (9000 x *g*, 30 min at 4 °C).

### Deuterium labeling of KRAS

Fifty milliliters of ZYM-20050-IO in a 250 mL baffled flask was inoculated with an ice chip from a *V. natriegens* glycerol stock. This seed culture was incubated overnight at 30°C and shaken at 250 rpm (1” throw). The following morning, the OD_600_ of the overnight culture was measured and used to inoculate 50 mL of ZYM-20050-IO in a 250 mL baffled flask with a starting OD_600_ of 0.3 and incubated at 30 °C, 250 rpm (1” throw), until reaching an OD_600_ of 1.5 (~ 1 h). Fifty mL of ModM9-1.5IO D_2_O (ModM9 adjusted to 1.5% w/v Instant Ocean™ and made with 100% D_2_O) was added to the flask (for 50% D_2_O in 100 mL of culture) and the 100 mL resulting culture transferred to a 500 mL baffled flask and the culture grown until OD_600_ = 1.5 (~ 1 h). One hundred mL of ModM9-1.5IO D_2_O was added to the culture (for 75% D_2_O in 200 mL of culture), transferred to a one-liter baffled flask and the culture grown until OD_600_ = 1.5 (~ 1.5 h). The cells were harvested by centrifugation (3900 x *g*, 15 min at room temperature) and resuspended in 100% ModM9 + 15 g/L NaCl in 100% D_2_O, and 250 mL aliquots were placed in four, one-liter baffled shake flasks. The cultures were grown to OD_600_ = 0.5, protein expression induced with the addition of 1 mM IPTG, and the incubator temperature shifted to 25 °C for overnight incubation. Cells were harvested by centrifugation (9000 x *g*, 30 min at 4 °C).

The protocols for deuterium labeling in *E. coli* were described previously [24, 25]. Briefly, an overnight culture was grown in MDAG medium [20] and washed cells from this overnight were used to inoculate LB Miller medium. After 1 h growth, an equal volume of 100% D_2_O ModM9 is added and after another 1 h growth this was repeated to achieve an overall four-fold increase in culture volume and a final D_2_O percentage of 75%. After 2 h growth in this condition, the cells were harvested by centrifugation (3500 x *g*, 30 min at room temperature), resuspended in the final production volume of 90% D_2_O ModM9, grown to an OD_600_ of 0.5, and protein expression induced with 0.5 mM IPTG. The culture was then incubated overnight at 25 °C and cells were harvested by centrifugation (9000 x *g*, 30 min at 4 °C).

### Cell lysis

Cells were lysed using a Microfluidics 110-EH (Microfluidics, Westwood, MA) by resuspending the cells in 10 mL of lysis buffer per 1000 OD_600_ of the final culture and processing at 13,000 PSI for two passes (10,000 PSI for *E. coli*). For RAF1 proteins, Benzonase™ Nuclease HC (cat # 71205-3, Millipore Sigma) was added at 250 units/L of culture and RNase (Qiagen) was added at 350 units/L of culture. Clarification of *V. natriegens* lysates is best achieved using ultra-centrifugation (104,000 x *g*) for 30 min. Alternatively, high-speed centrifugation (~ 13,400 x *g*, as used for *E. coli*) for 90 min can be used, but care should be taken when collecting the clarified supernatant after high-speed centrifugation, as there is often a loose portion of the cell debris pellet than can slough off and decant with the supernatant. We have observed increased back-pressure during column loading when this occurs.

### Protein purification

Small-scale purification screening by immobilized metal affinity chromatography (IMAC) was performed as described previously [26]. Briefly, His-tagged target proteins expressed in 50 ml cultures were isolated from clarified lysates using modified pipette tips packed with IMAC resin (PhyTips, BioTage, Upsalla, Sweden). All steps of the purification were carried out in a 96-well format using an automated liquid handler (MEA, BioTage). Chromatography was analyzed by SDS-PAGE/Coomassie staining.

Large-scale protein purifications were performed at room temperature using NGC workstations (BioRad, Hercules CA). G-Hs.KRAS4b(1-169), GG-Hs.KRAS4b(2-169) and G-Hs.NRAS(1-169) proteins were purified as described [27] (Protein production section, page S-4, in Supporting Information). Briefly, the expressed proteins of the form His6-MBP-tev-RAS were purified from clarified lysates by IMAC, treated with His6-TEV protease to release the RAS protein, and the RAS protein separated from other components of the TEV protease reaction by a second round of IMAC. Proteins were further purified by gel-filtration chromatography in a buffer containing 20 mM HEPES, pH 7.3, 150 mM NaCl, 2 mM MgCl_2_, and 2 mM TCEP. The peak fractions containing pure protein were pooled, flash frozen in liquid nitrogen, and stored at -80 °C.

Hs.RAF1(52–192) was purified as described previously for RAF1(52–188) [28]. Briefly, the approach is similar to that described above for RAS proteins, but with 500 mM NaCl and 10% glycerol used throughout the purification.

Nanobody purification was described previously [17]. Briefly, nanobodies expressed in the form of Nanobody-His6 were captured from clarified bacterial lysates by IMAC, concentrated and further purified and buffer exchanged via preparative size exclusion chromatography (SEC).

FLAG-Mm.AMHR2(18–142)-His6 was purified from the insoluble portion of the lysate using denaturing IMAC. The protein was then refolded on-column via SEC (manuscript in preparation). No major contaminants were detected by SDS-PAGE/Coomassie staining (Fig. 3B).

Of note is the additional time it takes to concentrate protein pools derived from *V. natriegens* expression material to the smaller volumes needed for preparative SEC. Generally, this takes approximately twice the time to process compared to the time needed to process similar pools from *E. coli* expression material. The cause of this phenomenon is unknown and the time to concentrate these pools can vary from lot to lot. *Vibrio* species are known to create biofilms and produce EPS under certain fermentation conditions [29, 30] which might be contributing to this phenomenon.

### Intrinsic GTPase measurement

The intrinsic GTPase rates for KRAS and NRAS were determined by the Phosphate Sensor assay [31]. A 2-fold, 8-point standard curve was generated using KH_2_PO_4_ with a starting concentration of 6 µM, with the last point being assay buffer (50 mM HEPES, pH 7.3, 150 mM KCl, 1.5 mM MgCl_2_, 5 mM DTT). RAS proteins (6 µM final concentration) were aliquoted into a 384-well microplate and the reaction was initiated by the addition of the Phosphate Sensor (4.5 µM final concentration). The phosphate standards were included in the plate. The plate was sealed to protect from the light and read from the bottom in the BioTek Synergy Neo2 (Ex 430/5, Em 450/5) every 2 min and 20 s for 8 h at 37 °C.

### Differential scanning fluorimetry (DSF)

DSF was performed on all samples as described in Niesen et al. [32]. Briefly, to attain Thermal Melting temperatures (Tm) 50 µL reactions were made by diluting protein samples to 11 µM in SEC buffer, and lastly adding Sypro Orange (Thermo Fisher Scientific). Protocols/data analysis were performed using BioRad CFX ManagerTM version 3.1 on a BioRad CFX96 instrument (Hercules, CA).

### Electrospray ionization mass spectrometry (ESI-MS)

All proteins purified in this work were analyzed by ESI-MS and found to be within 1–2 Da of the predicted mass. Mass spectrometry was done as previously described [33]. Briefly, samples were diluted to 0.1 mg/ml and 50 µL analyzed via liquid chromatography coupled on-line with mass spectrometry. High-resolution intact protein mass (MS1) spectra were acquired over a 600–2500 m/z window at 120,000 FT resolution (at 400 m/z) with an AGC target value of 3e + 06 and averaging 4 microscans. Spectra were analyzed by MagTran (Amgen Inc.).

## Results

### Optimization of transformation and growth

**Fig. 1:**
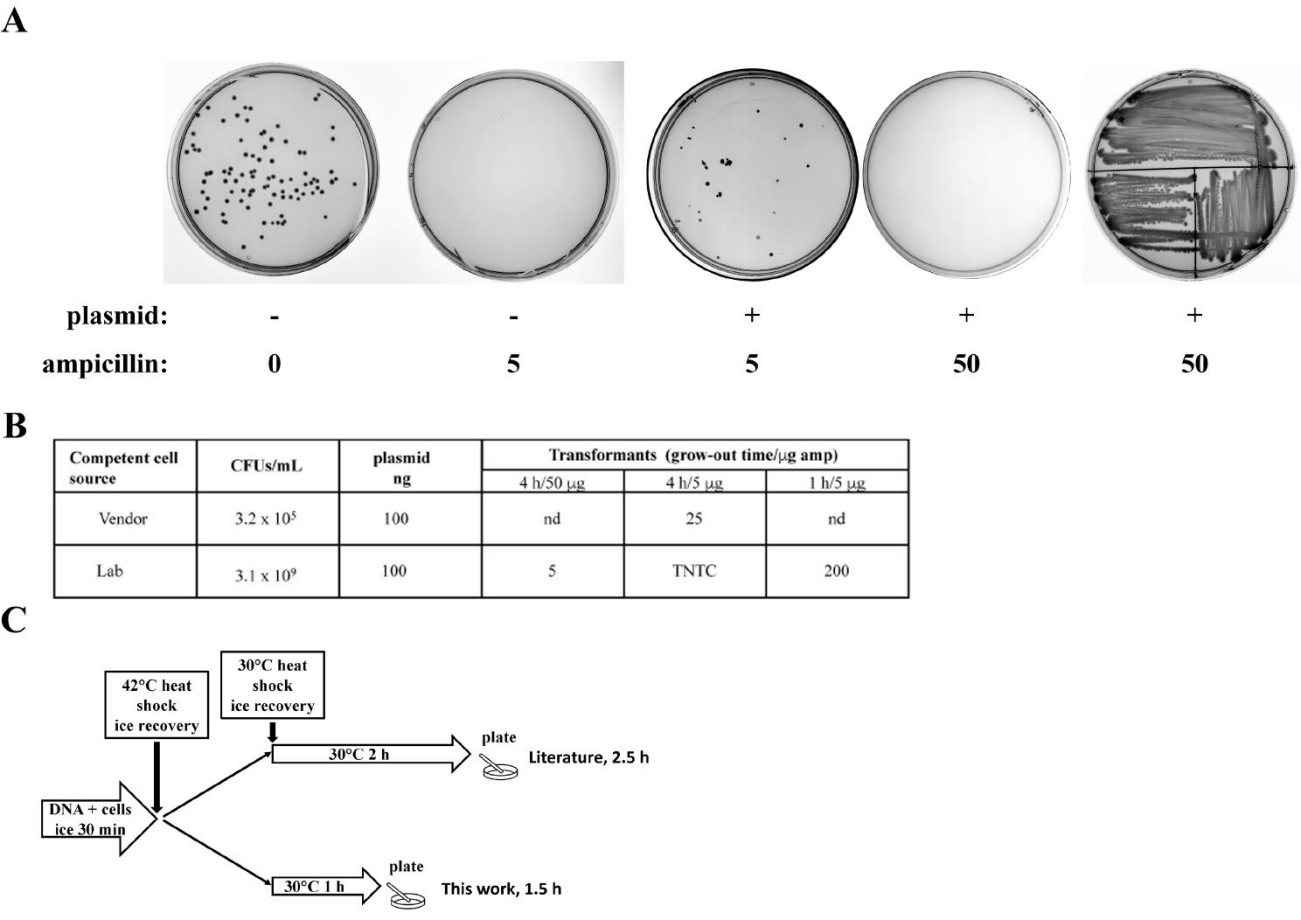
*V. natriegens* antibiotic resistance and transformation. (**A**) Photographs of agar plates spread with *V. natriegens*. Empty cells (‘-‘ plasmid) were plated on plates with zero or 5 ug/mL of ampicillin. *V. natriegens* transformation mixes (‘+’ plasmid, Amp^r^) were plated on plates with 5 µg/mL or 50 µg/mL ampicillin. A single isolated colony from the 5 µg/mL ampicillin plate was streak for isolation on a 50 µg/mL ampicillin plate (far right plate). (**B**) Analysis of competent cells. CFUs/mL were determined by plating dilutions of samples on non-selective plates. Transformants were plated after grow-out times indicated on plates with different ampicillin concentration. Representative colony counts from a transformation are listed. Nd – not determined, TNTC – too numerous to count. (**C**) Schematic of *V. natriegens* transformation protocols. Top path [4]. Bottom path, this work

Our initial attempts to transform *V. natriegens* resulted in insufficient transformants (frequently, no colonies arose). We traced the major source of the problem to higher ampicillin sensitivity than previously reported [4] when using our standard expression constructs which harbor the gene encoding ampicillin resistance (derived from pET-43). Lowering the ampicillin concentration from 50 µg/mL to 5 µg/mL resulted in sufficient colonies (Fig. 1A). Subsequent growth of isolated transformants on both solid and liquid media was possible at 50 µg/mL ampicillin, suggesting a delay or barrier in some aspect of establishing resistance compared to *E. coli*. Additionally, we found by using our in-house prepared chemically competent cells, we obtained more transformants as the cell density of our competent cells was higher than commercial sources (i.e. not through an increase in transformation efficiency) (Fig. 1B). We also modified the transformation protocol by eliminating the second heat shock step and shortening the grow out time to one hour. Note that the 4 h grow-out times noted in Fig. 1B were used only when troubleshooting the transformation protocol; one hour grow-out times were subsequently established as sufficient in our revised protocol. The changes we implemented shortened the protocol to 90 min with reduced manipulation (Fig. 1C).

**Fig. 2:**
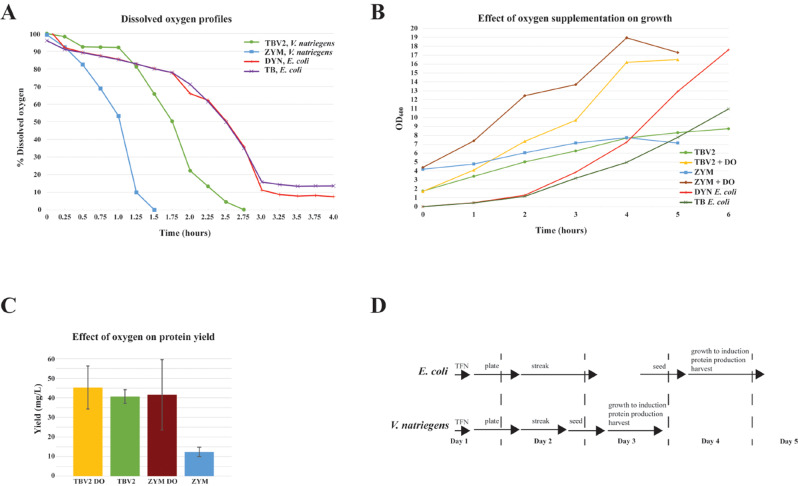
Growth parameters of *V. natriegens* and *E. coli* and schematic of protein production workflow (**A**) Dissolved oxygen profiles of *V. natriegens* and *E. coli* in standard growth media. Dissolved oxygen levels were measured of log phase cultures grown in protein production conditions. (**B**) Growths curves +/- oxygen supplementation of *V. natriegens* cultures in production media. Profiles of standard *E. coli* productions that typically do not receive supplemental oxygen are overlaid for comparison. (**C**) Protein yields for *V. natriegens* cultures depicted in panel B illustrating the effect of supplementing with dissolved oxygen. (**D**) Comparison of the workflow schematics for *E. coli* and *V. natriegens.* Arrows indicate approximate length of procedures (including incubation time) with the relevant procedure noted above the arrow. TFN – transformation, streak – T-streak for isolated colonies, seed – overnight culture from isolated colony (or glycerol stock, preferred for consistent subsequent culture growth)

The next step to evaluate *V. natriegens* as an alternative protein expression host was to develop a set of protocols that matched or exceeded the high cell densities and recombinant protein expression levels we routinely achieve with *E. coli* using our modifications [20] of the auto-induction protocols reported by the Studier lab [34]. In our *E. coli* system, cell densities of ~ 15–20 OD_600_ after overnight induction at 16 °C are routinely achieved. However, while *V. natriegens* did grow in modified versions of our auto-induction media ZYM-20052 (e.g. ZYM-20052 minus lactose and amended with 1.5% w/v NaCl; ZYM-20050-IO which also lacks lactose and is amended with 1.5% w/v Instant Ocean™ as described in the Methods), we encountered obstacles: (1) auto-induction of protein expression was not observed in ZYM-20052, (2) sub-culture growths for protein production from overnight seed cultures were inconsistent, and (3) IPTG-induced protein expression was reduced in the presence of Instant Ocean™. The lack of auto-induction may be due to the absence of a *lacY* gene (LacY for lactose transport) [35] and/or the absence of a *lacZ* gene (encoding beta-galactosidase to convert the lactose into the inducer allolactose) based on the genome sequence (https://www.ncbi.nlm.nih.gov/datasets/genome/GCF_001456255.1/). This may also bear on the poor IPTG-induced protein expression in the presence of Instant Ocean™ (point 3 above). While a specific link to Instant Ocean™ is not clear to us, the absence of *lacY* in *Vibrio* has been noted previously and was cited as a possible reason that induction of recombinant protein expression in *V*. *alginolyticus* required 5 mM IPTG [35]. To address the inconsistent growth of subcultures for protein production (point 2 above), we empirically determined that growing a 50 mL overnight seed culture (see Methods) in ZYM-20050-IO and subculturing the following day into TBV2 medium for protein production, gave consistent results in terms of the time to reach a desired OD_600_ and the growth kinetics of the subsequent subculture for protein production. Thus, subcultures grown in this way routinely lead to consistent and robust recombinant protein induction and allowed cell harvest 24 h after starting the overnight seed (see the summary of our protein production workflow depicted in the schematic in Fig. 2D). Conditions that did not provide consistent subculture growths include growing a 3 mL seed culture in a 24-well block, the lack of Instant Ocean™ in the overnight seed, and the presence of Instant Ocean™ in the subsequent protein production medium. The growth status of the culture seems to be implicated, but we have yet to determine the specific parameter(s) involved.

Due to the high growth rate and cell densities of our production cultures, we measured DO levels and found that under standard aeration conditions (no supplemental oxygen), cultures were below 20% DO within 1.25–2.0 h (Fig. 2A). We investigated the effect of supplementing oxygen to maintain 20% DO and while we did see an increase in the culture density (Fig. 2B), the protein yield was not substantially improved (Fig. 2C, data shown for cultures expressing His6-MBP-tev-GG-Hs.KRAS4b (2-169)) for this protein. This suggests that the amount of protein per cell was reduced. However, more investigation is required to determine the validity of this preliminary data as well as assessing whether expression of other proteins is similar. We prefer to use TBV2 medium with supplemental oxygen over ZYM-20052 (minus lactose, + 1.5% w/v NaCl) as our standard protein production medium for *V. natriegens* as it is a less complicated medium and the variation with ZYM-20052 + DO is larger (Fig. 2C). Additionally, the culture can be harvested within ~ 6–6.5 h from the inoculation of the production vessel from the overnight seed and within 24 h from inoculation of the overnight seed (Fig. 2D).

### Comparison of *V. natriegens* to *E. coli* in small-scale screening

With a reproducible and simple set of protocols in place for culturing *V. natriegens* and inducing protein expression, we compared the small-scale protein screening results between *E. coli* and *V. natriegens.* We routinely screen new constructs in a small-scale purification platform [26] for protein expression and purification in *E. coli* and baculovirus-infected insect cells. We added *V. natriegens* to the bacterial portion of this screen as the same plasmids can be used in both hosts. We discovered several cases where *V. natriegens* was the preferred expression host based on the results of this small-scale screening.

#### Anti-Müllerian hormone receptor type II (AMHR2) is expressed in *V. natriegens* but not in *E. coli*

**Fig. 3:**
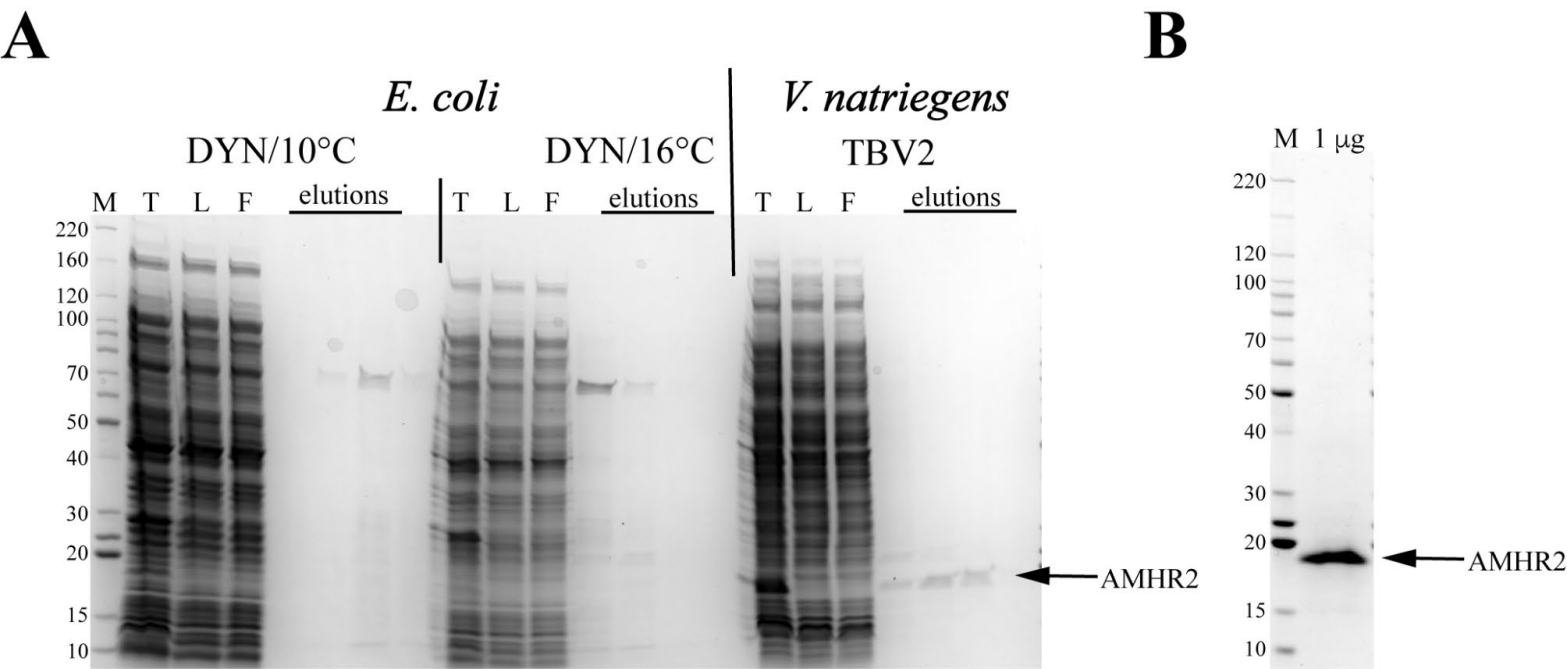
Small-scale screening comparison of FLAG-Mm.AMHR2(18–142)-His6 in *E. coli* and *V. natriegens* and final protein. (**A**) Representative results of small-scale purification screens from three conditions (two from *E. coli* and one from *V. natriegens*) analyzed by SDS-PAGE/Coomassie staining. M- protein standards (kDa), T – total lysate, L – column load, F – column flow through. (**B**) Final FLAG-Mm.AMHR2(18–142)-His6 purified from *V. natriegens* after scale-up expression, IMAC in denaturing condition, and SEC refolding (manuscript in preparation). One microgram of final protein was analyzed by SDS-PAGE/Coomassie staining

AMHR2 is a candidate for therapeutic treatment of epithelial ovarian carcinoma, the most prevalent form of ovarian cancer in the United States [36]. Specifically, the extracellular domain of murine AMHR2 (AMHR2-ED) has been used in murine vaccination studies with the goal of developing a therapeutic that induces preemptive immunity. The published protocol expresses the extracellular domain (amino acids 18–142) as FLAG-Mm.AMHR2(18–142)-His6. We were unable to replicate the published *E. coli* production result despite screening multiple expression conditions (our standard conditions of Dynamite 16 °C and additional conditions of LB/37 °C, Studier’s autoinduction ZYM20052/20 °C, Dynamite/37 °C, and Dynamite/10 °C). Two representative expression analyses (*E. coli*) and small-scale purification results are depicted in Fig. 3A. We then choose to compare our most common expression conditions in *E. coli*, Dynamite 16 °C [20], which often improves protein expression in our hands, to our recently adopted *V. natriegens* system. In Fig. 3A, no expression of FLAG-Mm.AMHR2(18–142)-His6 was detected in our standard condition of Dynamite 16 °C [20] whereas expression was strong, albeit mostly insoluble, in *V. natriegens*. This result allowed the development of a refolding protocol for the purification of AMHR2 (manuscript in preparation).

#### Small-scale screening suggests *V. natriegens* is superior to *E. coli* for production of neutralizing nanobodies to SARS CoV-2 spike

**Fig. 4:**
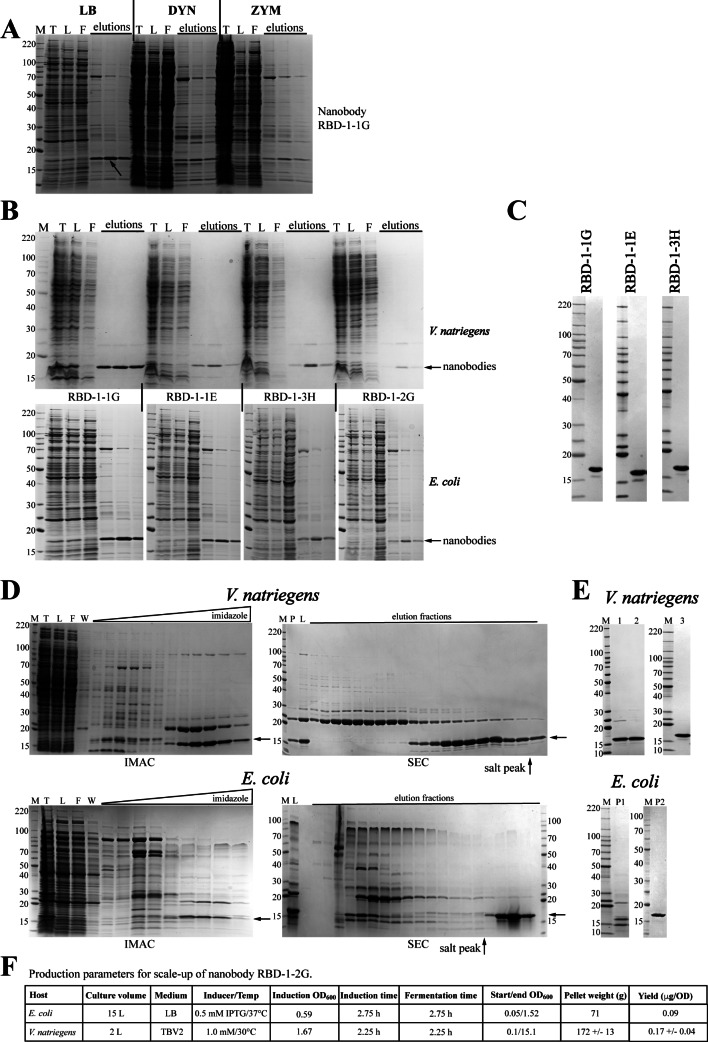
Comparison of *E. coli* and *V. natriegens* nanobody protein screening and production. (**A**) SDS-PAGE/Coomassie analysis of small-scale screening purification of nanobody RBD-1-1G expressed in *E. coli* under three standard conditions. M- protein standards (kDa), T – total lysate, L – column load, F – column flow through. (**B**) SDS-PAGE/Coomassie staining analysis of small-scale screening purification of four nanobodies expressed in *V. natriegens* (top) and *E. coli* (bottom). (**C**) Final protein analysis by SDS-PAGE/Coomassie staining of final proteins purified from scale-up expression in *V. natriegens*. One microgram of each final lot was loaded. (**D**) Scale-up purification of nanobody RBD-1-2G; comparison between *V. natriegens* (top panels) and *E. coli* (bottom panels). IMAC Load (L) is the soluble portion of the lysate, SEC load is the concentrated pool from IMAC step. W – column wash. (**E**) SDS-PAGE/Coomassie-stained gel of final nanobody RBD-1-2G preparations. One microgram of each preparation (three from *V. natriegens*, two from *E. coli*) was loaded. (**F**) Representative production parameters for RBD-1-2G productions

Our lab was tasked with producing neutralizing nanobodies to SARS CoV-2 spike for the National Center for Advancing Translational Research during the pandemic in 2020 [17]. The initial screen (Fig. 4A) was performed in *E. coli* and assessed three of our standard expression protocols: LB at 37 °C, Dynamite at 16 °C (DYN), and autoinduction (ZYM) at 20 °C. The results of small-scale IMAC screening from these expression materials indicated that LB at 37 °C was the preferred expression condition based on the quantity and purity of the protein as visualized by SDS-PAGE/Coomassie staining. However, we also evaluated our newly adopted (at the time) *V. natriegens* expression system as the purity of the protein from *E. coli* was not optimal. Parallel small-scale IMAC purification screening of the four nanobodies, each expressed in both *E*. *coli* (LB at 37 °C based on previous screen) and *V. natriegens*, are shown in Fig. 4B. The SDS-PAGE/Coomassie staining analysis indicated that while the nanobodies can be purified from both *E. coli* and *V. natriegens*, protein purity was higher from *V. natriegens*.

Interestingly, we noted the presence of a ~ 75 kDa contaminant in the elution fractions from *E. coli* purified proteins (see the elution lanes in the small-scale analyses in Figs. 3A and 4A, and 4B). Based on previous experiments, we believed this to be the *E. coli* chaperone DnaK. However, subsequent analysis of these small-scale elution fractions by ESI-MS identified the contaminant as the well-known IMAC binding contaminant ArnA [37]. A search of the *V. natriegens* genomic sequence did not reveal ArnA homologs which correlates with our observations that no contaminant of this size is detected by SDS-PAGE/Coomassie staining in the *V. natriegens* materials (Fig. 4B).

#### Scale-up of Nanobody RBD-1-2G: less aggregation and higher yield in *V. natriegens*

Three of the four nanobodies were subsequently purified from *V. natriegens* scale-up cultures (2 L) at a high level of purity (Fig. 4C) as expected from the small-scale screen and with acceptable yields in the range of 5.3 to 36.6 mg/L (Table 1). However, nanobody RBD-1-2G, was the least promising of the nanobodies in both systems based on the small-scale screening results (Fig. 4B). As this was the most promising of the nanobodies in terms of its neutralizing activity [17] it was critical to generate this reagent. Thus, as a direct comparison of the expression systems at larger scale, we expressed and purified RBD-1-2G from both systems. We chose the 2-liter scale for *V. natriegens* as the rich medium of TBV2 supported high cell densities, and the 15-liter scale for *E. coli* as LB medium would provide only limited cell mass. As indicated in Table 1, both *E. coli* productions were low yield and only one produced high purity protein as precipitation of the nanobody in the second preparation reduced the purity (sample P1 in Fig. 4E). In contrast, the purifications of RBD-1-2G from *V. natriegens* were more successful in terms of purity (Fig. 4, panels D and E) and yield (Table 1), with the added benefits of a 2-liter fermentation rather than 15 L. Intact mass analysis of the final proteins (15,594 Da for protein purified from both expression systems, compared to a predicted 15,597 Da) revealed no differences between the proteins produced.

Close inspection of the SEC steps from the scale-up productions (Fig. 4D), reveals a possible explanation for the poor yield from *E. coli.* The *E. coli* expressed nanobody eluted after the salt peak suggesting the nanobody interacted with the SEC resin while the nanobody expressed in *V. natriegens* largely eluted prior to the salt peak. This suggests a substantial qualitative difference between the nanobody purified from the two expression systems. Additionally, when the protein from the *E. coli* production was pooled from the post-salt peak SEC elution fractions, dialyzed, and concentrated, a precipitate formed. This further supports our hypothesis that the nanobody is, relative to *E. coli* produced protein, properly folded when expressed in *V. natriegens.* When comparing the fractions pooled from the SEC SDS-PAGE analysis (Fig. 4D) to the appearance of the final protein that remained in solution (*E. coli*-purified sample P1 in Fig. 4E), it can be deduced that the nanobody likely comprised the largest proportion of the precipitate. The subsequent second production from *E. coli* also eluted in the post-salt peak fractions during SEC. However, in that second production, the final material after dialysis was not concentrated and no precipitation was observed (see final sample, P2, in Fig. 4E). Regardless, the yield was still very low compared to that from *V. natriegens* (Table 1). Representative production parameters for both systems are shown in Fig. 4F.

### Comparison of *V. natriegens* and *E. coli* in scale-up production of RAS proteins

#### Yield of small GTPase KRAS4b is higher from *V. natriegens*

**Fig. 5:**
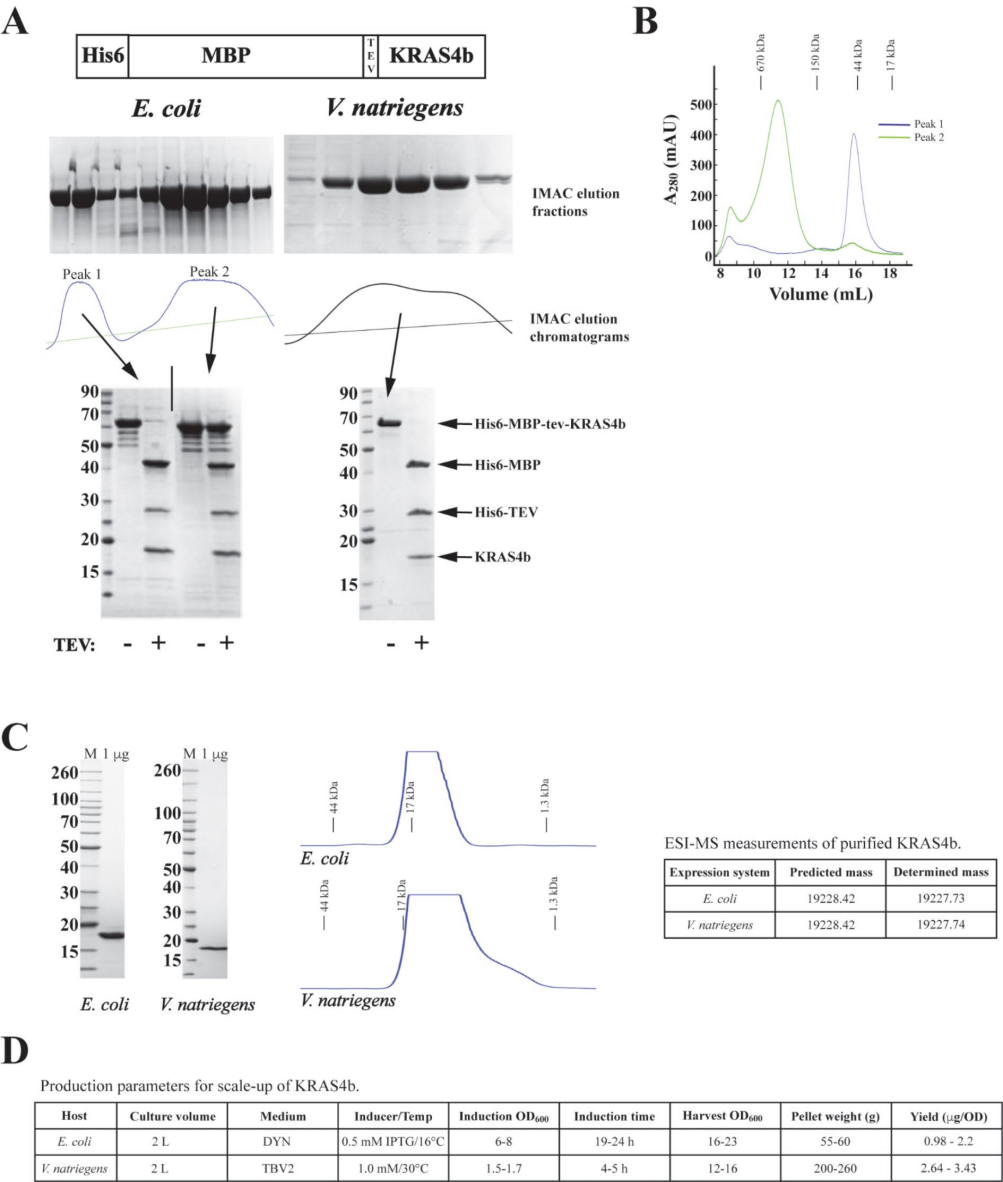
Comparison of the scale-up production of GG-Hs.KRAS4b(2-169) from *E. coli* and *V. natriegens*. (**A**) Top – schematic of expressed protein and SDS-PAGE/Coomassie analysis of IMAC elution fractions aligned with the A_280_ trace from the chromatograms. Bottom - TEV protease treatment of the resolved peaks (denoted by arrows) from the IMAC is analyzed by SDS-PAGE/Coomassie staining. (**B**) Overlaid analytical size exclusion chromatography (ANSEC) A_280_ traces from separate runs of Peak 1 and Peak 2 IMAC elution pools from *E. coli* purification in panel A. Elution of SEC standards are noted. (**C**) Representative QC of final RAS proteins from the two expression systems, left to right: SDS-PAGE/Coomassie-stained gel analysis, A280 trace of ANSEC, intact mass data from ESI-MS. (**D**) Representative production parameters for KRAS4b productions

Our lab produces a wide variety of proteins to support the RAS Initiative at the Frederick National Laboratory (https://frederick.cancer.gov/initiatives/ras-initiative). A significant proportion of that work is the production of four isoforms of the small GTPase family: HRAS, KRAS4a, KRAS4b, and NRAS. While the full-length proteins (~ 189 amino acids) have a C-terminal sequence that is involved in membrane localization/binding known as the hyper variable region, the reagent most used in our biochemical and structural experiments consist of the highly conserved amino acids of the GTPase domain (typically, amino acids 1-169, and commonly referred to as the G-domain). It was our work in producing the G-domain of KRAS4b, the protein that we have the most experience producing, that revealed a striking example of differential protein folding between the *E. coli* and *V. natriegens* expression systems. This was initially observed when comparing the chromatographic separation of the initial IMAC capture step for our most common expression construct, His6-MBP-tev-GG-Hs.KRAS4b(2-169) [24, 38, 39]. Figure 5A depicts the pertinent elution fractions (SDS-PAGE/Coomassie staining analysis and A_280_ trace) and the subsequent TEV protease treatment of pooled fractions. The elution profile of the *E. coli* preparation displays two large, resolved peaks (Peak 1 elution peak at ~ 140 mM imidazole from both systems and Peak 2 elution peak at ~ 250 mM imidazole from the *E. coli* system) that are confirmed by the SDS-PAGE/Coomassie staining analysis. Much of the *E. coli*-purified material is found in the later eluting Peak 2. This material is particularly resistant to TEV protease digestion (bottom SDS-PAGE/Coomassie stained gels in Fig. 5A) and migrates as a large species in an analytical SEC column (Fig. 5B) suggesting that this fraction is largely a soluble aggregate. The quantitatively smaller amount of material in Peak 1 is completely digested by TEV protease. In contrast, the same protein elutes as a single peak during IMAC when purified from *V. natriegens* and this protein is almost completely digested by TEV protease (Fig. 5A) and migrates as a monomer in SEC (Fig. 5B). Given that the yield for this protein is not dramatically different between the two systems (Table 1 indicates an approximate 40% yield increase for this protein in *V. natriegens*), the inference is that, while *V. natriegens* produces less total soluble fusion protein than *E. coli*, the proportion of correctly folded protein is higher leading to the higher yield from *V. natriegens*. The final proteins from both systems pass QC checks for SDS-PAGE/Coomassie staining purity, migration on SEC, and intact mass analysis (Fig. 5C). Useful attributes that are highlighted by the production parameters listed in Fig. 5D, include the shorter induction time of 4–5 h for *V. natriegens* and the incubation temperature of 30 °C. These two points are important attributes of the *V. natriegens* system to consider as no cooling incubation is required (preliminary experiments in our lab suggest lower temperature may reduce recombinant expression in *V. natriegens*) and the fermentation time is shortened to ~ 4 h versus the typical overnight induction at cooler temperature commonly used for *E. coli*.

#### Yield of small GTPase Hs.NRAS(1-169) is higher from *V. natriegens*

**Fig. 6:**
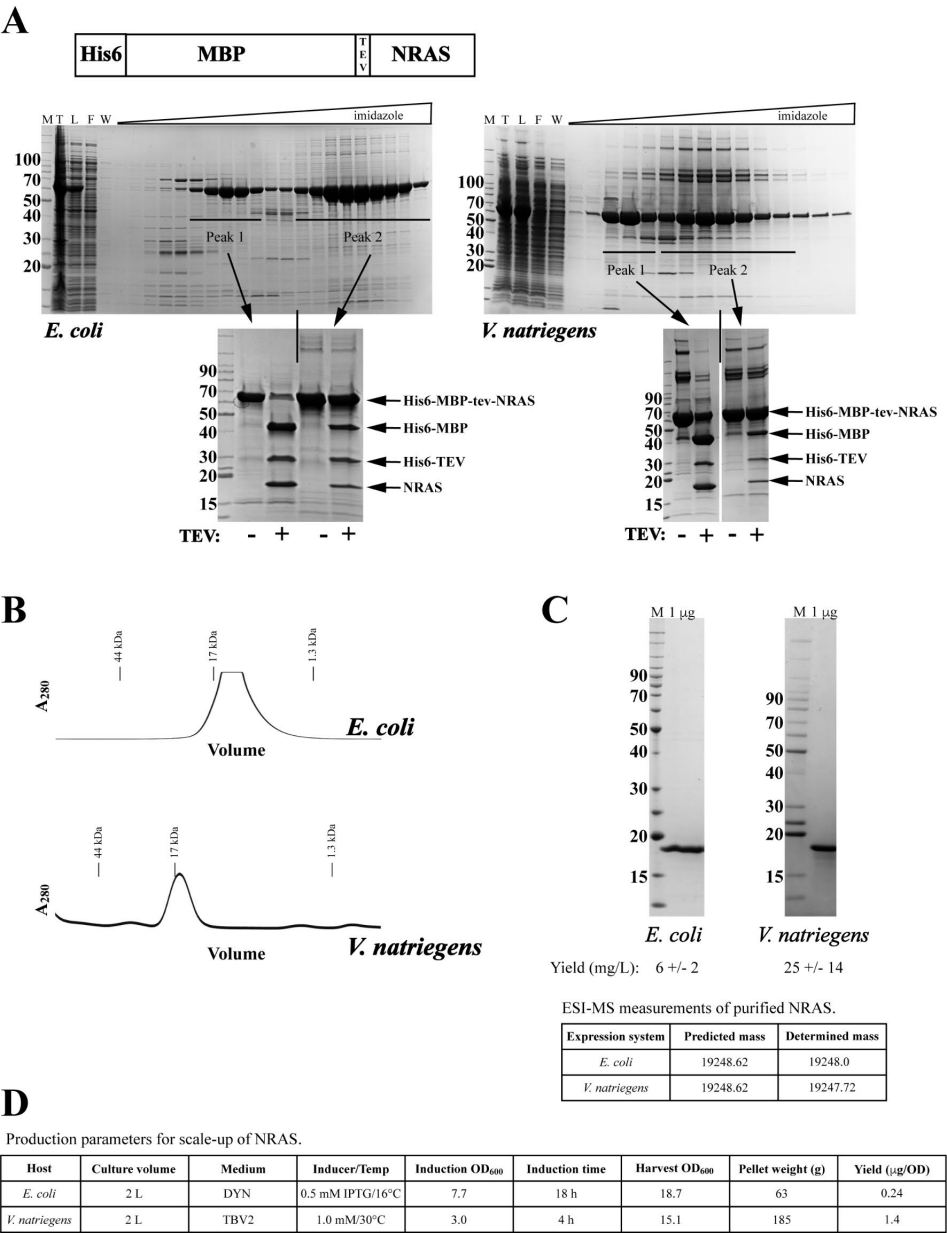
Comparison of the scale-up production of Hs.NRAS(1-169) from *E. coli* and *V. natriegens*. (**A**) Top – schematic of expressed protein. Bottom - SDS-PAGE/Coomassie analysis of IMAC elution fractions from representative purifications. SDS-PAGE/Coomassie analysis of TEV protease digestions of pooled fractions from designated peaks are shown below the IMAC analysis. (**B**) Representative A_280_ traces from preparative SEC of NRAS proteins. Elution of SEC standards are noted. (**C**) QC of final proteins from the two expression systems: SDS-PAGE/Coomassie-stained gel analysis and intact mass data from ESI-MS. (**D**) Representative production parameters for NRAS productions

We observed a similar phenomenon when producing the G-domain from the RAS isoform NRAS which has 92% sequence identity with the KRAS4b G-domain. In Fig. 6A, the shift to a higher percentage of IMAC elution Peak 1 relative to Peak 2 can be seen in the SDS-PAGE/Coomassie-stained gel analyses of parallel productions from the two expression systems. In this case, the shift was not as complete as observed in *V. natriegens* for KRAS4b, yet the increase in yield is approximately 2-fold (Table 1). The mid-points of elution for the peaks were slightly earlier for the material from *V. natriegens* than for *E. coli* (160 and 255 mM imidazole for Peaks 1 and 2, respectively, from *E. coli* compared to 145 and 205 mM imidazole for Peaks 1 and 2, respectively, from *V. natriegens*). From both systems, the later eluting Peak 2 protein was more recalcitrant to TEV digestion. Final proteins derived from the IMAC Peak 1 material from both systems passed the QC checks of migration on preparative SEC (Fig. 6B), SDS-PAGE/Coomassie staining purity (Fig. 6C), and intact mass analysis (Fig. 6C). Production parameters in Fig. 6D again point out two benefits of the *V. natriegens* fermentation: no need for cooling of the fermentation and shorter induction times compared to *E. coli*.

### Comparison of the GTPase activity of RAS proteins from *V. natriegens* and *E. coli*

To assess if the activity of the proteins produced from *V. natriegens* was similar to those made from *E. coli*, we measured the GTPase activity of KRAS4b and NRAS purified from the two systems by using a phosphate sensor assay. This assay uses the release of phosphate as a surrogate for GTPase activity [31]. Measured rates were similar in both cases: KRAS4b (*V. natriegens* 70 µM/min vs. *E. coli* 83 µM/min); NRAS (*V. natriegens* 71 µM/min vs. *E. coli* 73 µM/min).

### Improved yields of RAF1 kinase CR1 domain from *V. natriegens*

**Fig. 7:**
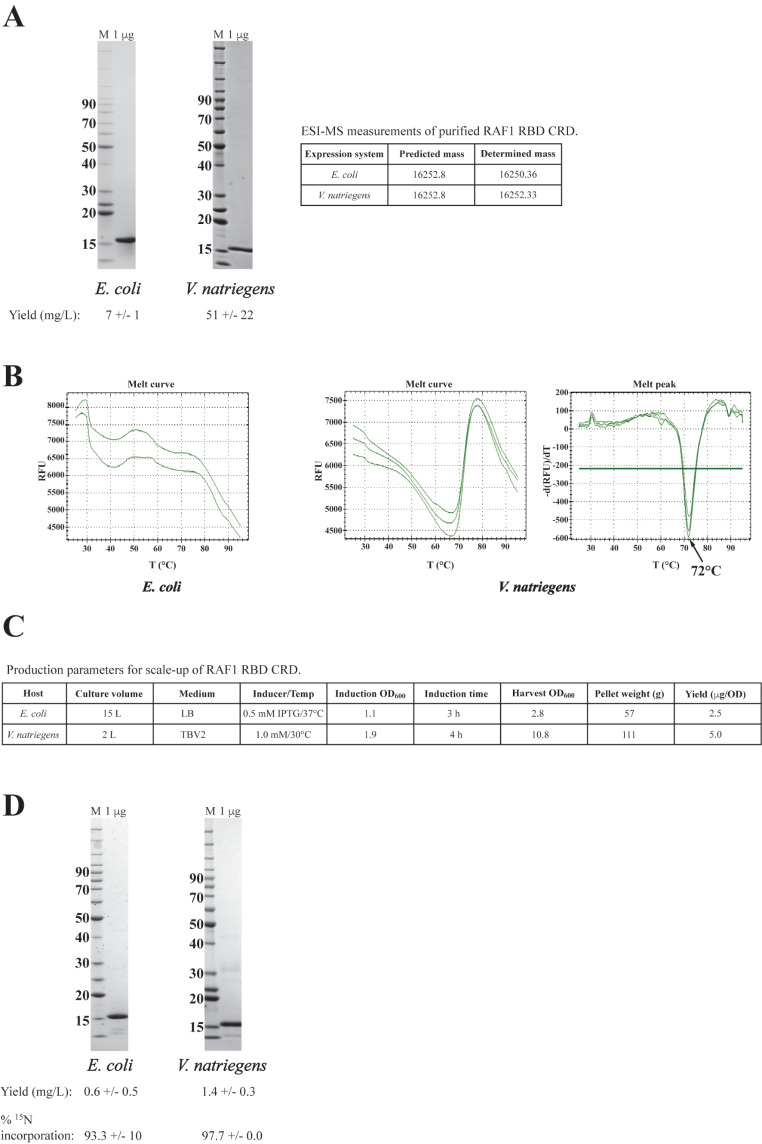
Comparison of scale-up productions of RAF1(52–192) from *E. coli* and *V. natriegens*. (**A**) SDS-PAGE/Coomassie-stained gel analysis, yield, and intact mass data from ESI-MS for representative final proteins from the two systems. (**B**) Representative Tm analysis data from the final proteins from each system. (**C**) Representative production parameters for RAF1(52–192) productions. (**D**) SDS-PAGE/Coomassie stain analysis of representative final samples of ^15^N labeled RAF1(52–192), yield and ^15^N incorporation data (*n* = 3 for each system)

The observations from our work with the small GTPases, led us to attempt to express and purify Hs.RAF1(52–192), also known as RAF1 CR1, from *V. natriegens*. RAF1 CR1 is domain of RAF1 kinase comprised of the RAS binding domain (RBD) and the cysteine rich domain (CRD) [28] which play roles in RAS binding and localization to the inner membrane of the cell, respectively. RAF1 plays a pivotal role in RAS-driven cancers as it is the continuous activation of RAF1 kinase by constitutively active oncogenic RAS proteins that drives many cancers [40]. While the smaller RAF1 RBD is a relatively easy protein to produce, the presence of the cysteine rich domain in the RAF1 CR1 construct, reduces the yield to below 10 mg/L from *E. coli* (See Fig. 7A and C, and Table 1). However, yields from the *V. natriegens* system are significantly higher (Fig. 7A and C, and Table 1) and a Tm was measured for the protein (72–74 °C), which is not routinely possible with the *E.* coli produced protein (Fig. 7B). The Tm of the RAF1 CR1 construct has not been reported in the literature to our knowledge, however, most laboratories use only the RAF1 RBD. The inability to obtain a measurable Tm is thought to be due to partially unfolded protein and/or the presence of hydrophobic amino acids at or near the surface of the protein. The fluorescent dyes used in DSF, fluoresce when bound to hydrophobic moieties, and this produces high initial background fluorescence which masks any subsequent increase in fluorescence during the assay as the protein is ‘melted’ during the experiment to expose the hydrophobic core [32].

### Isotopic labeling in *V. natriegens* is comparable to *E. coli*

A considerable roadblock in our lab is achieving practical and inexpensive production of isotopically labeled proteins. When purification yields drop below one milligram/liter, the cost of isotopically labeled proteins becomes prohibitive due to the larger fermentation volume (and thus reagent cost). As others have shown recently, *V. natriegens* can also be used to produce isotopically labelled proteins [6, 15]. Thus, we explored producing isotopically labelled Hs.RAF1(52–192) in *V. natriegens*, hoping to reduce costs due to the higher yield. This was investigated by comparing the yield from ^15^N isotopic labeling productions between *E. coli* and *V. natriegens* (Table 1; Fig. 7D). While modest, the increase from 0.6 mg/L to 1.4 mg/L does merit the consideration of producing these more expensive reagents in the *V. natriegens* system. The percentage of ^15^N incorporation for both systems was high: 97.7% +/- 0 for *V. natriegens*, 93.3% for *E. coli*). Similarly, we investigated the possibility of producing deuterated proteins in *V. natriegens*. Comparable yields were achieved between the two systems as indicated in Table 1. We calculated the percentage of non-exchangeable hydrogens that were deuterated and again comparable results were achieved with an average of 79.1% +/- 1 (*n* = 3) deuteration for the *V. natriegens* lots and 78.6% (*n* = 2) deuteration for the *E. coli* lots.

**Table 1:** Proteins produced in this work

Expressed protein	Purified protein	Yield (mg/L)*	
		*E. coli*	*V. natriegens*
FLAG-Mm.AMHR2(18–142)-His6	FLAG-Mm.AMHR2(18–142)-His6	nd^1^	7.0 +/- 3.3
Nanobody RBD-1-1G	RBD-1-1G	nd^2^	7.5
Nanobody RBD-1-1E	RBD-1-1E	nd^2^	36.6
Nanobody RBD-1–3 H	RBD-1–3 H	nd^2^	5.3
Nanobody RBD-1-2G	RBD-1-2G	0.1, 0.004	2.5 +/- 0.4
His6-MBP-tev-GG-Hs.KRAS4b(2-169)	GG-Hs.KRAS4b(2-169)	29 +/- 7	41 +/- 4
His6-MBP-tev-G-Hs.NRAS(1-169)	G-Hs.NRAS(1-169)	6 +/- 2	25 +/- 14
His6-MBP-tev-RAF1(52–192)	Hs.RAF1(52–192)	7 +/- 1	51 +/- 22
His6-MBP-tev-RAF1(52–192) ^15^N	Hs.RAF1(52–192) ^15^N	0.6 +/- 0.5	1.4 +/- 0.3
His6-MBP-tev-G-Hs.KRAS4b(1-169) D_2_O	G-Hs.KRAS4b(1-169) D_2_O	2.7, 1.9^3^	3.8 +/- 0.6

* When a standard deviation is reported, a minimum of three independent productions were performed

^1^ Due to the lack of expressed protein, no scale up was possible

^2^ Due to the poor prognosis at small-scale screen, no scale up was attempted in *E. coli*. A single production was produced in *V. natriegens*

^3^ Result from two independent productions

## Discussion

We present findings and protocols that should help interested laboratories add *V. natriegens* to their recombinant protein production workflow. Specifically, our work defines a set of seed and production media and protocols that builds on our previously published work in *E. coli* [20] (with modifications to Studier’s auto-induction studies), that are key to a reproducible system. Additionally, our observations of sensitivity to levels of ampicillin lower than previously reported and optimized transformation protocol were key to adopting the system. The longer grow-out period after heat shock during transformation reported in the literature is unusual, especially considering the fast growth rate of *V. natriegens*. By lowering the ampicillin concentrations used to select for transformants, we may have overcome some limitation that subsequently allowed us to shorten the grow-out time. Another critical factor was our finding of the effects that Instant Ocean™ can have on culture growth, reproducibility, and protein expression. We suspect some of these results might be due to differences with the approaches and techniques or inherent physiology of *V. natriegens*, rather than a difference between isolates of *V. natriegens*, but that remains to be investigated.

Once in place, the potential of the *V. natriegens* system, with its fast growth rate, was apparent. The ability to generate a cell pellet within 24 h from seed culture inoculation, substantially changes the timelines, and thus throughput, of the laboratory (Fig. 2D): pushing the bottleneck to equipment preparation rather than fermentation. While supplemental oxygen is necessary to achieve this timeline, the improvements in expression, solubility and yield reported here do not require this (e.g. Figure 2C indicates that maintaining 20% dissolved oxygen in the TBV2 medium does not clearly increase protein yields).

Overall, we found using *V. natriegens* as an alternative expression host for recombinant protein expression improved several projects in the lab with little increase in cost or effort. Also, in our laboratory, we routinely express proteins overnight at 16 °C. This is unnecessary for *V. natriegens* expression and may be deleterious based on initial observations. This aspect of *V. natriegens* culture may be useful for laboratories without the ability to maintain lower temperatures during growth.

In some cases, the *V. natriegens* expression system was responsible for the success of a protein production project (i.e. the target protein could not be produced from *E. coli*, as was the case for AMHR2, Fig. 3A) and/or allowed a stalled project to proceed by reducing fermentation volume/costs compared with *E. coli* (as was the case for several NRAS projects due to the large increase in yield, Fig. 6). Similarly, increased yield allowed the consideration of isotopically labelled difficult-to-express proteins that would have been prohibitive to produce in *E. coli*, as was the case to produce ^15^N RAF1(52–192) and deuterated KRAS4b (both reported here).

However, the most intriguing aspect of the *V. natriegens* system, from our point of view, is the apparent improvement in protein folding suggested by our data. Several results reported here support this hypothesis: (1) different elution profile of nanobodies on SEC indicating interaction with the column for the nanobody RBD-1-1G (2) absence/reduction of the soluble aggregate species of His6-MBP-tev-RAS fusions and (3) obtaining a Tm measurement for the RAF1 CR1 domain. These observations span a range of protein families and expression levels, suggesting the possibility of fundamental differences between *E. coli* and *V. natriegens* that might be exploited to improve protein folding in both systems. Whether the mechanism of this phenomenon is due to increased chaperones, elevated levels of ribosomes, and/or some other factor(s), is not known. These enhancements to the production of the difficult to produce proteins reported here, illustrate the potential benefits from adding this easy to use, genetically amenable, and microbiologically diverse *V. natriegens* system to the toolbox for recombinant protein production.

## Conclusions

*V. natriegens* can be an important addition to a protein expression laboratory. While not a replacement for *E. coli*, *V. natriegens* produces some proteins at higher yield and with less aggregation. Incorporating *V. natriegens* alongside existing small- and large-scale *E. coli* project workflows is relatively simple and requires no additional equipment or reagents. In so doing, we have been able to complete several projects heretofore stalled in the standard *E. coli* expression system due to poor yield and/or protein aggregation. Given the diversity of proteins represented in these projects from the limited number of protein families we have investigated, it seems likely that *V. natriegens* will be useful in the production of additional proteins.

## Data Availability

All data generated or analysed during this study are included in this published article.

## Abbreviations

DSF: Differential scanning fluorimetry
ESI-MS: Electrospray ionization mass spectrometry
IMAC: Immobilized metal affinity chromatography, SEC – size exclusion chromatography
